# Predictors of Attrition and Academic Success of Medical Students: A 30-Year Retrospective Study

**DOI:** 10.1371/journal.pone.0039144

**Published:** 2012-06-18

**Authors:** Silvija Maslov Kruzicevic, Katarina Josipa Barisic, Adriana Banozic, Carlos David Esteban, Damir Sapunar, Livia Puljak

**Affiliations:** 1 Health Center Split, Split, Croatia; 2 Department of Anatomy, Histology and Embryology, University of Split School of Medicine, Split, Croatia; St Michael’s Hospital, University of Toronto, Canada

## Abstract

**Aim:**

To determine attrition and predictors of academic success among medical students at University of Split, Croatia.

**Methods:**

We analysed academic records of 2054 students enrolled during 1979–2008 period.

**Results:**

We found that 26% (533/2054) of enrolled students did not graduate. The most common reasons for attrition were ‘personal’ (36.4%), transfer to another medical school (35.6%), and dismissal due to unsatisfactory academic record (21.2%). Grade point average (GPA) and study duration of attrition students were significantly associated with parental education. There were 1126 graduates, 395 men and 731 women. Their average graduation GPA was 3.67±0.53 and study duration 7.6±2.44 years. During 5-year curriculum only 6.4% (42/654) of students graduated in time, and 55% (240/472) of students graduated in time after curriculum was extended to 6 years. Variables predicting whether a student will graduate or not were high school grades, entrance exam score and year of enrollment. Significant predictors of graduation grades were high school grades and entrance exam score. Entrance exam score predicted length of studying.

**Conclusion:**

Preadmission academic qualifications and year of enrollment predict academic success in medical school. More attention should be devoted to high attrition.

## Introduction

Enrolling into medical school represents the start of a demanding and stressful period for students. Despite a multitude of social, academic, and emotional stressors, most students successfully cope with a complex new life role and achieve academic success. Other students are less able to successfully manage this transition and, sooner or later, decide to withdraw themselves, or face dismissal by medical school.

Attrition rates are one of important indicators being used to measure university performance. In part, this reflects the fact that student attrition represents an inefficient use of resources if students who leave the school before graduating cannot use in the labor market whatever human capital they have gained during their courses [1].

A medical student’s training is a considerable personal investment of time and money for the student and student’s parents, and in many countries it is also a major investment of governmental money [2]. For the benefit of both students and society it is therefore important to minimize the rate of attrition by optimizing the student selection procedure and having robust mechanisms to identify and support students with academic difficulties [2]. Knowing predictors of academic failure and success is also important for medical schools that are trying to ensure high completion rates and develop support mechanisms for students with inadequate performance. A recent study showed that those who perform poorly in the early years of medical school, for whatever reason, might be at an increased risk for subsequent professional misconduct [3]. By studying factors associated with academic failure and success among medical students, it could be possible to detect an early warning signs of academic failure.

Analysis of attrition and predictors of academic success might yield multiple long-term benefits. However, while studies about selecting medical students are numerous, this is not the case for attrition and detecting potential failures. Studies of attrition and identification of students who may struggle both academically and personally are important to enable evidence-based selection and support services at medical schools.

For this reason, we decided to make a 30-year retrospective analysis to explore significant preadmission and academic factors that may contribute to attrition, as well as academic success. We also analyzed characteristics of successful and unsuccessful students of this School.

## Methods

### Ethics Statement

The retrospective data collection was approved by the Ethics Committee of the University of Split School of Medicine. When filling application and enrollment forms, students confirm with a signature that their data can be archived and used for analyses. Information about grades are stored in each student file. As this study used deidentified student analyses, informed consent was waived.

### Setting

Independent University of Split School of Medicine (USSM) was founded in 1997, but the entire medical studies in Split, Croatia were conducted since 1979, when the school was subsidiary of the University of Zagreb School of Medicine. Croatian educational system uses grades 1–5 for appraising knowledge, where 1 indicates failure and 5 is the highest grade. In 2005 the curriculum and study rules were changed according to the Bologna Accord.

### Data

Retrospective data collection was performed at the archives of the USSM. The following data were used: high school grade point average (GPA), entrance exam scores (EES), GPA at the end of the medical school, study duration and whether a student graduated or not. For those students who enrolled into the USSM, but did not graduate in this School for any reason, we collected information on reason for attrition, type of high school, education of parents, grades obtained before leaving the school, names of failed courses, number of times that students failed those subjects and we calculated average grade that student has had before leaving the school.

### Statistical Analysis

Variables were studied using analytical software SPSS 15.0 (SPSS Inc., Chicago, IL, USA). Associations between variables were tested using Pearson’s correlation and Student’s t-test. Data from descriptive statistics were expressed as mean ± standard deviation (SD), or median and interquartile range. Multivariate regression analysis was used to identify independent predictors of attrition. Where appropriate, confidence interval (CI) was indicated. Statistical significance was set at p<0.05.

## Results

### Analysis of Attrition

We studied 30 successive generations of medical students, who enrolled the USSM from 1979 to 2008. Of 2054 enrolled students, 533 (26%) failed to graduate for various reasons; among them there were 215 men (40%) and 318 women (60%). Half of the attrition students were lost during the first (N = 269) year, followed by 23% (N = 120) in the second, 14% (N = 76) in the third, 9% (N = 49) in the fourth, 3.5% (N = 20) in the forth and 0.5% (N = 3) in the sixth study year. Student office divided reasons for attrition into three categories: voluntary withdrawal, transfer to another medical school, or dismissal by the School due to academic failure or absenteeism. Unspecified personal reasons and transfer to another medical school were the most common causes of attrition (Table 1).

**Table 1:** Causes of attrition.

Reasons	No. (%) of students	Average grade*	Average study duration in years (mean±SD)
Personal reasons	194 (36.4)	3.3	3.9±3.87
Dismissed due to poor academic performance	113 (21.2)	3.3	12.7±5.88
Transfer to another medical school	190 (35.6)	3.5	2.6±2.05
Died	7 (1.3)	3.6	3.7±2.50
Unspecified	29 (5.4)	3.4	18.5±4.95
Total	533 (100)	3.4	5.4±5.5

* Students who did not pass a single exam during their studies are excluded from this calculation (n = 153).

Length of studying of attrition students ranged from 0–24 years, as some students signed out shortly after enrollment, and some students neglected their studies until the School dismissed them. Average study duration among attrition students was 5.4±5.5 years. Table 1 shows differences in study duration based on reasons for attrition. We found statistically significant difference in study duration between groups of students with different reasons for attrition (F(4,484) = 250.7, p<0.001). The actual number of years lost by them in attending medical school amounted to 2684. In 2009, the cost of one student-year in this School was 8700 USD, which means that equivalent to more than 23 million USD in 2009 value was spent on students who did not graduate at this School.

Among attrition students, 29% (153/533) did not pass a single exam during their studies. For the remaining 71% of attrition students we calculated average grade point average (GPA) that they gained before dropping out, and it was 3.4 out of maximum 5.0 (Table 1). Students without a GPA studied significantly shorter than those who passed at least some exams: their study duration was 4.3 and 5.8 years, respectively (p<0.01).

Information about exams that attrition students took while still studying, revealed that the most commonly failed exams were Anatomy, Histology and Embryology (Table 2). During the first 30 years of the School, subject Anatomy and subject Histology and embryology were for 9 years united in a course Structure of the human body; therefore we pooled results from these three subjects, as visible in Table 2.

**Table 2:** Courses that attrition students took more than once.

Course	No. of students who failedthe course	Average number of times the exam was taken
Anatomy, Histology and Embryology	33	4.1
Medical physics and Biophysics	20	4.0
Biology	12	3.8
Physiology	8	6.4
Microbiology	2	6
Biochemistry	7	4.9
Pharmacology	2	6
Patophysiology	3	4.7
Basic neuroscience	5	6.6
Chemistry	2	3.5
Pathology	1	8
Immunology	1	2

Among 533 attrition students we studied those who had failed to pass at least one exam during the time they were studying. Some students had more problematic courses; 28 students failed 2 exams at least once, while 2 students failed 3 exams at least once. There were 36 students who failed one course, 28 who failed two courses, and 2 students who failed three courses during their studies.

GPA of attrition students was correlated with maternal (r = 0.18, p<0.01) and fraternal (r = 0.11, p<0.05) education. Attrition students’ length of studying was negatively correlated with maternal education (r = −0.17, p<0.01). Better high school education (i.e. in grammar school versus professional high school) was correlated with GPA (r = 0.12, p<0.05). Positive correlation was found between type of high school attended by the students, and grades in medical school (r = 0.21 p<0.05), meaning that students from grammar schools achieve better grades while studying.

### Predictors of Academic Failure or Success

During analyzed 30 years, 1126 students graduated, 395 men and 731 women, with a combined graduation GPA of 3.67 and average study duration 7.6 years (range: 5–25 years). Graduation GPA was correlated with high school GPA (r = 0.27, p<0.01) and EES (r = 0.34, p<0.01). Length of studying was negatively correlated with graduation GPA (r = −0.39, p<0.01) and admission test scores (r = −0.38 p<0.01).

Linear regression analysis was conducted in order to test whether entrance exam and high school GPA were predictive of academic success. Significant predictors of success defined with medical school GPA were high school GPA (b = 0.19, p<0.01) and entrance exam score (b = 0.29, p<0.01). This model in total explained 27% of variance.

Gender did not significantly contribute to the model. Entrance exam score also significantly predicted length of studying (b = −0.12, p<0.01), while high school GPA was not significant predictor for length of studying. This model explained 15% of variance.

Direct logistic regression was performed to assess the impact of a number of factors on the likelihood that students would graduate or not graduate. The model contained four independent variables (gender, high school grades, entrance exam grades and year of enrolment). The full model containing all predictors was significant, (χ^2^ (4.1459) = 55.66, p<0.001) indicating that the model was able to distinguish between students who graduated and did not graduate. The model as a whole explained between 4.3% (Cox and Snell R square) and 6.3% (Nagelkerke R squared) of the variance of academic failure. Three of the independent variables made a unique statistically significant contribution to the model (entrance exam scores, high school scores and year of enrolment). The strongest predictor was year of enrolment and odds ratio of 1.03 (CI = 1.02–1.05).

## Discussion

We found that variables predicting whether a student will graduate or not were high school grades, entrance exam score and year of enrollment. Significant predictors of academic success, defined with graduation grades, were high school grades and entrance exam score. Entrance exam score also predicted length of studying.

Attrition occurs when a student fails to graduate from a medical school. During analyzed 30-year period, attrition amounted to 26% of students. This attrition rate is very high, and it did not change from a 2001 report [4]. Study of American medical students between 1987 and 2005 showed that about 80% of them graduated on time, and attrition was less than 4% [5], lower than previously reported attrition of 8.7% in 1949–1958 period [6]. At Leeds School of Medicine the attrition rate between 1983 and 1992 was 14% [7]. One of the reasons for such a high attrition could be that most of the students in Croatia do not pay for their university tuitions; those are funded by the government instead. Under those circumstances, students may not have a feeling of obligation that comes with a personal financial investment in education.

There are different reasons for attrition, both academic and non-academic. In our study, the most common reasons for not graduating in this School were poorly defined ‘personal’ reasons, and transfer to another medical school. Reasons for these transfers should be investigated in more detail, to see why almost 10% of students enrolled in this School choose to continue their studies elsewhere. Since this is a regional School, it is possible that transfer students enrolled at the USSM because they were not able to enroll medical school of their first choice, with plans to transfer later to another medical school [8].

In our recent nation-wide study, applicants to medical schools in Croatia indicated that their choice of a medical school was mostly based on the quality of school and its reputation; however, when we compared in which city they live and what school they choose, it turned out that majority of the respondents chose the geographically closest school [9].

Attrition students in total spent 2684 years officially studying, which is a huge waste of resources. Therefore, we explored preadmission and academic records of attrition students, to see whether there are factors that may help in recognizing students at risk of academic failure. Identification of students at-risk is particularly worthy because interventions have been described that may offer support to these students and improve their academic performance [10], [11].

Factors associated with academic success in our study were parental education, high school grades and entrance exam scores. Correlation with entrance exam scores was found for both graduation grades and length of studying as indicators of academic success. Enrollment in the USSM has always been based on objective academic criteria, with a formula that combined high school grades and entrance exam scores. Throughout the School’s history, this formula was fairly consistent, with one third of the points for enrollment coming from high school grades, while two thirds of the points were derived from the result of entrance exam. Our results show that this decision was justified, as entrance exam is correlated with both indicators of academic success.

However, selection policies and students’ intellectual abilities are not solely responsible for attrition. Poor teaching and poor evaluation are also exceedingly important [12]. Surely, not all of the dropouts should have been graduated, as some of them probably lack the ability or other personally qualities needed to enter the medical practice. On the other hand, if methods of recruiting, selection, teaching and evaluating can be improved, then we can expect that higher proportion of our entrants can become adequate physicians [13]. Therefore, findings of the present study prompted us to provide several recommendations. Once the students are admitted, there should be instruments to identify students at risk for academic failure. Since our study showed that 50% of the attrition happens during freshmen year, an interview with a student counselor could be scheduled for all students after the first semester to identify and support those at risk for attrition.

Furthermore, record keeping at the School should be modernized, with electronic databases including all student-related information; this would allow ongoing data analysis and monitoring of students and their performance. Also, more information should be collected and included in the files of attrition students. While searching for these data, in many student files we found reasons for withdrawal that were vague and not informative enough, such as ‘personal’ reasons, ‘desire to try different studies’, etc. According to the Student Office, some of the students decided to drop out themselves after finding out that the School will dismiss them due to inadequate academic record, but this is not indicated in the student files.

In conclusion, measures should be implemented to reduce student attrition and increase number of students graduating on time. This can be aided by using predictors of academic outcomes to recognize students at risk, and provide them with adequate support.
